# 1,4-Diazo­niabicyclo­[2.2.2]octane tetra­bromidocadmate(II) monohydrate

**DOI:** 10.1107/S160053680902813X

**Published:** 2009-07-22

**Authors:** Kong Mun Lo, Seik Weng Ng

**Affiliations:** a Department of Chemistry, University of Malaya, 50603 Kuala Lumpur, Malaysia

## Abstract

The metal atom in the anion of the title salt, (C_6_H_14_N_2_)[CdBr_4_]·H_2_O, shows a slightly distorted tetra­hedral coordination. The water mol­ecule is involved in three hydrogen bonds, *viz.* one N—H⋯O and two O—H⋯Br, and an N—H⋯Br inter­action consolidates the three-dimensional network.

## Related literature

For other ammonium tetra­bromidocadmates, see: Al-Far & Ali (2008 ▶); Battaglia *et al.* (1991 ▶); Chen *et al.* (2006 ▶); Geselle & Fuess (1994 ▶); Hatano *et al.* (2008 ▶); Ishihara *et al.* (2002 ▶, 2006 ▶); Ravikumar *et al.* (1995 ▶); Waskowska (1994 ▶); Zhang & Fang (2005 ▶).
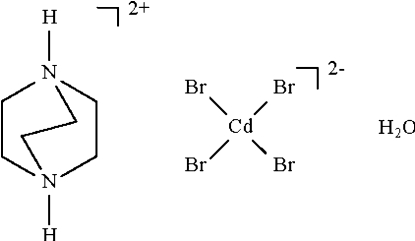


## Experimental

### 

#### Crystal data

(C_6_H_14_N_2_)[CdBr_4_]·H_2_O
*M* *_r_* = 564.25
Orthorhombic, 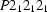

*a* = 8.6323 (1) Å
*b* = 11.8736 (2) Å
*c* = 13.5619 (2) Å
*V* = 1390.05 (4) Å^3^
*Z* = 4
Mo *K*α radiation
μ = 13.04 mm^−1^
*T* = 296 K
0.30 × 0.15 × 0.05 mm

#### Data collection

Bruker SMART APEX diffractometer
Absorption correction: multi-scan (*SADABS*; Sheldrick, 1996 ▶) *T*_min_ = 0.111, *T*_max_ = 0.562 (expected range = 0.103–0.521)
10779 measured reflections
2451 independent reflections
2267 reflections with *I* > 2σ(*I*)
*R*_int_ = 0.034

#### Refinement

*R*[*F* ^2^ > 2σ(*F* ^2^)] = 0.057
*wR*(*F* ^2^) = 0.187
*S* = 1.36
2451 reflections
128 parameters
H-atom parameters constrained
Δρ_max_ = 2.21 e Å^−3^
Δρ_min_ = −2.09 e Å^−3^
Absolute structure: Flack (1983 ▶), 1021 Friedel pairs
Flack parameter: 0.14 (3)

### 

Data collection: *APEX2* (Bruker, 2008 ▶); cell refinement: *SAINT* (Bruker, 2008 ▶); data reduction: *SAINT*; program(s) used to solve structure: *SHELXS97* (Sheldrick, 2008 ▶); program(s) used to refine structure: *SHELXL97* (Sheldrick, 2008 ▶); molecular graphics: *X-SEED* (Barbour, 2001 ▶); software used to prepare material for publication: *publCIF* (Westrip, 2009 ▶).

## Supplementary Material

Crystal structure: contains datablocks global, I. DOI: 10.1107/S160053680902813X/tk2504sup1.cif

Structure factors: contains datablocks I. DOI: 10.1107/S160053680902813X/tk2504Isup2.hkl

Additional supplementary materials:  crystallographic information; 3D view; checkCIF report

## Figures and Tables

**Table 1:** Hydrogen-bond geometry (Å, °)

*D*—H⋯*A*	*D*—H	H⋯*A*	*D*⋯*A*	*D*—H⋯*A*
O1w—H11⋯Br4^i^	0.84	2.72	3.15 (3)	113
O1w—H12⋯Br1^ii^	0.84	2.83	3.65 (3)	167
N1—H1⋯Br1	0.86	2.92	3.568 (11)	134
N2—H2⋯O1w	0.86	2.04	2.80 (2)	146

Symmetry codes: (i) 
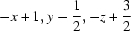
; (ii) 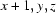.

**Fig. 1.**
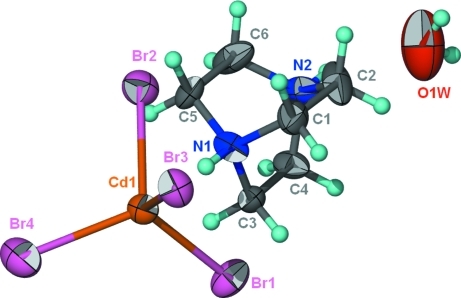
Thermal ellipsoid plot (Barbour, 2001) of [C6H14N2][CdBr4].H2O at the 50% probability level. Hydrogen atoms are drawn as spheres of arbitrary radius.

## supplementary crystallographic information

### Experimental

Triethylenediammonium dibromide was prepared from the reaction of triethylenediamine (1 g, 1.68 mmol) with bromine (1:2) in the presence of excess hydrobromic acid.  To this was added cadmium chloride hemipentahydrate (0.38 g, 1.68 mmol) in ethanol (50 ml).  The mixture was heated for an hour.  The filtered solution when allow to evaporate slowly yielded colorless crystals.

### Refinement

C- and N-bound H atoms were placed at calculated positions (C–H 0.97Å and N–H 0.86 Å) and were treated as riding on their parent atoms with *U*(H) set to 1.2*U*_eq_(C, N). The water-bound H atoms were placed in chemically sensible positions on the basis of hydrogen bonding interactions but were not refined.

The final difference Fourier map had a peak 0.2 Å from Cd1 and a hole 0.4 Å from Br4.

### Crystal data

**Table:**

(C_6_H_14_N_2_)[CdBr_4_]·H_2_O	*F*(000) = 1048
*M**_r_* = 564.25	*D*_x_ = 2.696 Mg m^−^^3^
Orthorhombic, *P*2_1_2_1_2_1_	Mo *K*α radiation, λ = 0.71073 Å
Hall symbol: P 2ac 2ab	Cell parameters from 6547 reflections
*a* = 8.6323 (1) Å	θ = 2.8–28.2°
*b* = 11.8736 (2) Å	µ = 13.04 mm^−^^1^
*c* = 13.5619 (2) Å	*T* = 296 K
*V* = 1390.05 (4) Å^3^	Block, colorless
*Z* = 4	0.30 × 0.15 × 0.05 mm

### Data collection

**Table:**

Bruker SMART APEX diffractometer	2451 independent reflections
Radiation source: fine-focus sealed tube	2267 reflections with *I* > 2σ(*I*)
graphite	*R*_int_ = 0.034
ω scans	θ_max_ = 25.0°, θ_min_ = 2.3°
Absorption correction: multi-scan (*SADABS*; Sheldrick, 1996)	*h* = −10→10
*T*_min_ = 0.111, *T*_max_ = 0.562	*k* = −14→14
10779 measured reflections	*l* = −16→16

### Refinement

**Table:**

Refinement on *F*^2^	Secondary atom site location: difference Fourier map
Least-squares matrix: full	Hydrogen site location: inferred from neighbouring sites
*R*[*F*^2^ > 2σ(*F*^2^)] = 0.057	H-atom parameters constrained
*wR*(*F*^2^) = 0.187	*w* = 1/[σ^2^(*F*_o_^2^) + (0.1*P*)^2^ + 5*P*] where *P* = (*F*_o_^2^ + 2*F*_c_^2^)/3
*S* = 1.36	(Δ/σ)_max_ = 0.001
2451 reflections	Δρ_max_ = 2.21 e Å^−^^3^
128 parameters	Δρ_min_ = −2.09 e Å^−^^3^
0 restraints	Absolute structure: Flack (1983), 1021 Friedel pairs
Primary atom site location: structure-invariant direct methods	Flack parameter: 0.14 (3)

### Fractional atomic coordinates and isotropic or equivalent isotropic displacement parameters (Å^2^)

**Table:**

	*x*	*y*	*z*	*U*_iso_*/*U*_eq_	
Cd1	0.25482 (11)	0.47127 (8)	1.00019 (6)	0.0410 (3)	
Br1	0.3099 (2)	0.28309 (13)	0.91553 (12)	0.0596 (5)	
Br2	0.4870 (2)	0.59802 (15)	0.95680 (13)	0.0579 (5)	
Br3	0.2467 (2)	0.46741 (14)	1.19150 (10)	0.0552 (4)	
Br4	0.0032 (2)	0.56945 (19)	0.95508 (17)	0.0741 (6)	
O1W	1.151 (3)	0.265 (2)	0.6666 (18)	0.144 (9)	
H11	1.1965	0.2150	0.6336	0.216*	
H12	1.2016	0.2746	0.7189	0.216*	
N1	0.6148 (13)	0.4081 (10)	0.7757 (7)	0.040 (3)	
H1	0.5272	0.4215	0.8034	0.048*	
N2	0.8660 (14)	0.3708 (12)	0.6978 (13)	0.063 (4)	
H2	0.9523	0.3577	0.6682	0.076*	
C1	0.725 (2)	0.3684 (14)	0.8530 (12)	0.056 (4)	
H1A	0.6860	0.3008	0.8846	0.067*	
H1B	0.7396	0.4260	0.9029	0.067*	
C2	0.882 (2)	0.3435 (18)	0.7975 (16)	0.086 (8)	
H2A	0.9647	0.3877	0.8264	0.104*	
H2B	0.9083	0.2645	0.8042	0.104*	
C3	0.5912 (16)	0.3214 (11)	0.7016 (12)	0.045 (3)	
H3A	0.5121	0.3449	0.6551	0.054*	
H3B	0.5577	0.2519	0.7326	0.054*	
C4	0.7409 (17)	0.3035 (13)	0.6494 (9)	0.047 (3)	
H4A	0.7308	0.3262	0.5810	0.056*	
H4B	0.7679	0.2242	0.6510	0.056*	
C5	0.6697 (19)	0.5139 (11)	0.7291 (11)	0.044 (3)	
H5A	0.6781	0.5729	0.7783	0.052*	
H5B	0.5968	0.5382	0.6790	0.052*	
C6	0.824 (2)	0.4924 (13)	0.6836 (17)	0.066 (5)	
H6A	0.9016	0.5400	0.7143	0.079*	
H6B	0.8206	0.5100	0.6138	0.079*	

### Atomic displacement parameters (Å^2^)

**Table:**

	*U*^11^	*U*^22^	*U*^33^	*U*^12^	*U*^13^	*U*^23^
Cd1	0.0398 (5)	0.0445 (6)	0.0387 (5)	0.0007 (4)	0.0034 (5)	−0.0059 (4)
Br1	0.0813 (11)	0.0441 (8)	0.0535 (8)	0.0002 (8)	0.0191 (7)	−0.0081 (7)
Br2	0.0570 (9)	0.0650 (10)	0.0515 (8)	−0.0032 (8)	−0.0029 (7)	0.0044 (7)
Br3	0.0546 (8)	0.0682 (10)	0.0428 (7)	0.0061 (9)	0.0040 (7)	0.0035 (6)
Br4	0.0606 (11)	0.0862 (13)	0.0756 (12)	0.0076 (10)	−0.0051 (9)	−0.0244 (10)
O1W	0.150 (18)	0.153 (19)	0.129 (16)	0.073 (17)	0.022 (14)	0.012 (15)
N1	0.037 (5)	0.063 (7)	0.020 (5)	−0.003 (5)	0.003 (4)	−0.009 (5)
N2	0.029 (6)	0.057 (8)	0.104 (12)	−0.002 (5)	0.019 (7)	−0.038 (8)
C1	0.067 (11)	0.051 (9)	0.051 (8)	0.005 (8)	−0.023 (8)	0.003 (7)
C2	0.089 (14)	0.071 (12)	0.099 (15)	0.039 (11)	−0.064 (13)	−0.051 (11)
C3	0.036 (6)	0.031 (7)	0.069 (9)	0.000 (5)	−0.002 (7)	0.000 (6)
C4	0.045 (7)	0.056 (8)	0.040 (6)	−0.008 (7)	−0.005 (7)	−0.014 (6)
C5	0.059 (9)	0.026 (6)	0.045 (7)	0.002 (6)	0.007 (7)	0.000 (6)
C6	0.066 (11)	0.040 (9)	0.091 (12)	−0.011 (7)	0.035 (10)	−0.017 (8)

### Geometric parameters (Å, °)

**Table:**

Cd1—Br4	2.540 (2)	C1—H1A	0.9700
Cd1—Br1	2.557 (2)	C1—H1B	0.9700
Cd1—Br2	2.574 (2)	C2—H2A	0.9700
Cd1—Br3	2.596 (2)	C2—H2B	0.9700
O1W—H11	0.84	C3—C4	1.49 (2)
O1W—H12	0.84	C3—H3A	0.9700
N1—C3	1.453 (18)	C3—H3B	0.9700
N1—C5	1.484 (18)	C4—H4A	0.9700
N1—C1	1.491 (18)	C4—H4B	0.9700
N1—H1	0.8600	C5—C6	1.49 (2)
N2—C2	1.40 (3)	C5—H5A	0.9700
N2—C4	1.495 (18)	C5—H5B	0.9700
N2—C6	1.50 (2)	C6—H6A	0.9700
N2—H2	0.8600	C6—H6B	0.9700
C1—C2	1.58 (3)		
			
Br4—Cd1—Br1	116.87 (7)	N2—C2—H2B	109.8
Br4—Cd1—Br2	110.01 (7)	C1—C2—H2B	109.8
Br1—Cd1—Br2	105.29 (7)	H2A—C2—H2B	108.2
Br4—Cd1—Br3	103.05 (7)	N1—C3—C4	107.9 (11)
Br1—Cd1—Br3	116.00 (7)	N1—C3—H3A	110.1
Br2—Cd1—Br3	105.05 (6)	C4—C3—H3A	110.1
H11—O1W—H12	107.7	N1—C3—H3B	110.1
C3—N1—C5	110.5 (10)	C4—C3—H3B	110.1
C3—N1—C1	110.6 (12)	H3A—C3—H3B	108.4
C5—N1—C1	111.3 (12)	C3—C4—N2	110.0 (11)
C3—N1—H1	108.1	C3—C4—H4A	109.7
C5—N1—H1	108.1	N2—C4—H4A	109.7
C1—N1—H1	108.1	C3—C4—H4B	109.7
C2—N2—C4	111.8 (15)	N2—C4—H4B	109.7
C2—N2—C6	111.8 (15)	H4A—C4—H4B	108.2
C4—N2—C6	106.5 (14)	N1—C5—C6	108.5 (11)
C2—N2—H2	108.9	N1—C5—H5A	110.0
C4—N2—H2	108.9	C6—C5—H5A	110.0
C6—N2—H2	108.9	N1—C5—H5B	110.0
N1—C1—C2	105.7 (13)	C6—C5—H5B	110.0
N1—C1—H1A	110.6	H5A—C5—H5B	108.4
C2—C1—H1A	110.6	C5—C6—N2	109.1 (12)
N1—C1—H1B	110.6	C5—C6—H6A	109.9
C2—C1—H1B	110.6	N2—C6—H6A	109.9
H1A—C1—H1B	108.7	C5—C6—H6B	109.9
N2—C2—C1	109.5 (13)	N2—C6—H6B	109.9
N2—C2—H2A	109.8	H6A—C6—H6B	108.3
C1—C2—H2A	109.8		
			
C3—N1—C1—C2	60.9 (16)	C2—N2—C4—C3	57.5 (17)
C5—N1—C1—C2	−62.3 (16)	C6—N2—C4—C3	−64.8 (17)
C4—N2—C2—C1	−60.2 (17)	C3—N1—C5—C6	−63.7 (16)
C6—N2—C2—C1	59.0 (19)	C1—N1—C5—C6	59.6 (16)
N1—C1—C2—N2	2(2)	N1—C5—C6—N2	2(2)
C5—N1—C3—C4	58.4 (15)	C2—N2—C6—C5	−63 (2)
C1—N1—C3—C4	−65.3 (15)	C4—N2—C6—C5	59.1 (19)
N1—C3—C4—N2	6.0 (17)		

### Hydrogen-bond geometry (Å, °)

**Table:**

*D*—H···*A*	*D*—H	H···*A*	*D*···*A*	*D*—H···*A*
O1w—H11···Br4^i^	0.84	2.72	3.15 (3)	113
O1w—H12···Br1^ii^	0.84	2.83	3.65 (3)	167
N1—H1···Br1	0.86	2.92	3.568 (11)	134
N2—H2···O1w	0.86	2.04	2.80 (2)	146

Symmetry codes: (i) −*x*+1, *y*−1/2, −*z*+3/2; (ii) *x*+1, *y*, *z*.

## References

[bb1] Al-Far, R. & Ali, B. F. (2008). *J. Chem. Crystallogr.* **37**, 333–341.

[bb2] Barbour, L. J. (2001). *J. Supramol. Chem.* **1**, 189–191.

[bb3] Battaglia, L. P., Corradiab, B., Cariatif, K. & Koman, M. (1991). *Inorg. Chim. Acta*, **187**, 141–147.

[bb4] Bruker (2008). *APEX2* and *SAINT* Bruker AXS Inc., Madison, Wisconsin, USA.

[bb5] Chen, W.-T., Zeng, X.-R., Fang, X.-N., Li, X.-F. & Kuang, H.-M. (2006). *Acta Cryst.* C**62**, m571–m573. 10.1107/S010827010604131X 17148887

[bb6] Flack, H. D. (1983). *Acta Cryst.* A**39**, 876–881.

[bb7] Geselle, M. & Fuess, H. (1994). *Acta Cryst.* C**50**, 1582–1585.

[bb8] Hatano, N., Nakashima, M., Horiuchi, K., Terao, H. & Ishihara, H. (2008). *Z. Naturforsch. Teil B*, **63**, 1181–1186.

[bb9] Ishihara, H., Horiuchi, K., Gesing, T. M., Dou, S.-Q., Buhl, J. C. & Erk, P. (2002). *Z. Natursforsch.* **57**, 503–508.

[bb10] Ishihara, H., Koriuchi, K., Svoboda, I., Fuess, H., Gesing, T. M., Buhl, J. C. & Terao, H. (2006). *Z. Naturforsch. Teil B*, **61**, 69–72.

[bb11] Ravikumar, K., Venkata Lakshmi, N., Swamy, G. Y. S. K. & Chandra Mohan, K. (1995). *Acta Cryst.* C**51**, 1556–1558.

[bb12] Sheldrick, G. M. (1996). *SADABS* University of Göttingen, Germany.

[bb13] Sheldrick, G. M. (2008). *Acta Cryst.* A**64**, 112–122. 10.1107/S0108767307043930 18156677

[bb14] Waskowska, A. (1994). *Z. Kristallogr.* **209**, 750–754.

[bb15] Westrip, S. P. (2009). *publCIF* In preparation.

[bb16] Zhang, H. & Fang, L. (2005). *Acta Cryst.* E**61**, m101–m102.

